# Inhibition of acid sphingomyelinase by tricyclic antidepressants and analogons

**DOI:** 10.3389/fphys.2014.00331

**Published:** 2014-09-02

**Authors:** Nadine Beckmann, Deepa Sharma, Erich Gulbins, Katrin Anne Becker, Bärbel Edelmann

**Affiliations:** Department of Molecular Biology, Institute of Molecular Biology, University of Duisburg-EssenEssen, Germany

**Keywords:** ceramide, acid sphingomyelinase, antidepressants, amitriptyline, signaling

## Abstract

Amitriptyline, a tricyclic antidepressant, has been used in the clinic to treat a number of disorders, in particular major depression and neuropathic pain. In the 1970s the ability of tricyclic antidepressants to inhibit acid sphingomyelinase (ASM) was discovered. The enzyme ASM catalyzes the hydrolysis of sphingomyelin to ceramide. ASM and ceramide were shown to play a crucial role in a wide range of diseases, including cancer, cystic fibrosis, diabetes, Alzheimer's disease, and major depression, as well as viral (e.g., measles virus) and bacterial (e.g., *Staphylococcus aureus, Pseudomonas aeruginosa*) infections. Ceramide molecules may act in these diseases by the alteration of membrane biophysics, the self-association of ceramide molecules within the cell membrane and the ultimate formation of larger ceramide-enriched membrane domains/platforms. These domains were shown to serve the clustering of certain receptors such as CD95 and may also act in the above named diseases. The potential to block the generation of ceramide by inhibiting the ASM has opened up new therapeutic approaches for the treatment of these conditions. Since amitriptyline is one of the longest used clinical drugs and side effects are well studied, it could potentially become a cheap and easily accessible medication for patients suffering from these diseases. In this review, we aim to provide an overview of current *in vitro* and *in vivo* studies and clinical trials utilizing amitriptyline to inhibit ASM and contemplate possible future applications of the drug.

## Introduction

In the past, lipids were mainly known to be important for keeping cell shape. Since then, a lot of new information has changed our understanding of lipids and awarded them with new importance. According to current knowledge lipids are involved in many different signaling pathways, including cell survival, proliferation, and differentiation, but also in senescence and apoptosis. One major group of lipids involved in these pathways are the sphingolipids. A key enzyme in the sphingolipid pathway is acid sphingomyelinase (human: ASM, murine: Asm). This enzyme was shown to play a crucial role in many different diseases, for example in major depression, cancer, cystic fibrosis, and infectious diseases.

In this review, we will discuss the impact of lipid domain formation by ASM in these and other ASM-related diseases and go on to discuss the potential clinical benefits of inhibiting ASM in these conditions. To this end, we will address current studies employing ASM inhibitors, especially the functional ASM inhibitor amitriptyline, which is already clinically used as an antidepressant. We will critically assess the effectiveness of amitriptyline and also point out potential new applications in therapeutic treatment of diseases. Next to amitriptyline, we will also consider further treatment possibilities relying on the inhibition of ASM.

## Acid sphingomyelinase

Acid sphingomyelinase is an endo-lysosomal protein of 629 amino acids and with a molecular weight of 75/72 kDa or, after limited proteolysis during maturation, 57 kDa, respectively (Hurwitz et al., 1994a). The enzyme catalyzes the hydrolysis of sphingomyelin to phosphorylcholine and the second messenger ceramide with an optimum of pH at 5.0. The importance of ASM is evident in a lysosomal storage disorder called Niemann-Pick disease type A and B, in which sphingomyelin accumulates in the endo-lysosomal compartment due to ASM deficiency.

Two forms of ASM are described depending on their cellular localization (Takahashi et al., 2005; Jenkins et al., 2009, 2010; Milhas et al., 2010): a lysosomal ASM (L-SM) and a secretory ASM (S-SM). The two forms result from alternative trafficking of the precursor protein. Both forms can be distinguished by their glycosylation status: due to the Golgi-mediated transport to the plasma membrane, S-SM is N-glycosylated in a more complex manner compared to L-SMase (Schissel et al., 1998). Six glycosylation sites have been identified in the ASM protein and deglycosylation results in inactivation of the protein (Ferlinz et al., 1997). Further, S-SM activity depends on Zn^2+^ ions, L-SM has already bound Zn^2+^ ions and is therefore independent of exogenous ions (Schissel et al., 1996, 1998). In 2001, Grassmé and coworkers demonstrated for the first time that ASM activation is linked to translocation of the enzyme to the plasma membrane upon CD95 stimulation (Grassmé et al., 2001a). Since then, a large number of studies have analyzed the regulation of lysosomal ASM with respect to ASM translocation in combination with ASM activation in response to different stimuli (e.g., Grassmé et al., 2002; Abdel Shakor et al., 2004; Lacour et al., 2004; Rotolo et al., 2005; Dumitru and Gulbins, 2006; Zeidan and Hannun, 2007; Zhang et al., 2007; Perrotta et al., 2010; Edelmann et al., 2011; Li et al., 2012, 2013), but only a few studies have addressed the secreted form of the enzyme (Schissel et al., 1996, 1998; Wong et al., 2000; Jenkins et al., 2009) and these studies have been limited to cell models, plasma, or serum (Mühle et al., 2013).

ASM can be activated by various stimuli, e.g., reactive oxygen species, death receptors, irradiation, stress stimuli, or infections (for overview see Table 1). Different activation mechanisms have been described in this context. For example, ASM can be activated by phosphorylation: it has been demonstrated that protein kinase C delta (PKCδ) phosphorylates ASM at Ser508, resulting in immediate activation and translocation to the plasma membrane (Zeidan and Hannun, 2007; Zeidan et al., 2008). A further mechanism describes the proteolytic cleavage of ASM by caspase-7, resulting in increased activity (Edelmann et al., 2011). In addition, at least *in vitro* enzyme activity can be enhanced by the modification of a free, C-terminal cysteine (Cys629) and this activation mechanism seems to be essentially identical to the “cysteine switch” known for metalloproteases: in the low activity form, Cys629 is involved in the active site zinc coordination, impairing the enzymes ability for nucleophilic attack. Increased activity results from the absence of Cys629 in the coordination of the active site zinc atom (e.g., due to dimerization of ASM via this cysteine), which increases catalytic effectiveness (Qiu et al., 2003).

**Table 1:** **Overview of stimuli inducing acid sphingomyelinase and/or ceramide-enriched membrane platforms formation**.

**Stimulus**	**References**
**PATHOGENS**
*Listeria monocytogenes*	Utermöhlen et al., 2003
*Measles virus*	Gassert et al., 2009; Avota et al., 2011
*Mycobacterium avium*	Utermöhlen et al., 2008
*Neisseria gonorrhoeae*	Grassmé et al., 1997; Hauck et al., 2000
*Pseudomonas aeruginosa*	Grassmé et al., 2003a; Zhang et al., 2008
*Rhinoviruses*	Grassmé et al., 2005; Dreschers et al., 2007; Miller et al., 2012
*Salmonella typhimurium*	McCollister et al., 2007
*Sindbis virus*	Jan et al., 2000
*Staphylococcus aureus*	Esen et al., 2001
**CLUSTER OF DIFFERENTIATION MOLECULES**	
CD5	Simarro et al., 1999
CD14	Pfeiffer et al., 2001
CD20	Bezombes et al., 2004
CD28	Boucher et al., 1995
CD32 (FCγRII)	Abdel Shakor et al., 2004; Korzeniowski et al., 2007
CD38	Jia et al., 2008
CD40	Grassmé et al., 2002
CD95	Cifone et al., 1994; Gulbins et al., 1995; Cremesti et al., 2001; Perrotta et al., 2010
CD95-DISC	Kirschnek et al., 2000; Grassmé et al., 2001a,b, 2003b
CD253 (TRAIL)	Dumitru and Gulbins, 2006; Dumitru et al., 2007; Li et al., 2013
IL-1 receptor	Mathias et al., 1993
**SOLUBLE MOLECULES**
Platelet activating factor	Samapati et al., 2012; Predescu et al., 2013
Tumor necrosis factor	Schütze et al., 1992, 1994; Garcia-Ruiz et al., 2003; Edelmann et al., 2011; Ardestani et al., 2013
Visfatin	Boini et al., 2010a
**DRUGS AND OTHER STRESSES**
Cisplatin	Lacour et al., 2004; Zeidan et al., 2008
Cu^2+^-treatment	Lang et al., 2007
Doxorubicin	Dumitru et al., 2007
Heat damage	Chung et al., 2003
Ischemia-reperfusion injury	Yu et al., 2000
Oxidative stress	Zhang et al., 2007; Li et al., 2012
Oxygen radicals	Scheel-Toellner et al., 2004
UV-light	Zhang et al., 2001; Charruyer et al., 2005; Kashkar et al., 2005; Rotolo et al., 2005
γ-irradiation	Santana et al., 1996; Paris et al., 2001; Lee et al., 2011

### ASM-mediated formation of ceramide-enriched membrane domains

In 1972 Singer and Nicolson proposed the so called “fluid mosaic model,” describing the organization of biological membranes. They postulated a random distribution of lipids and proteins and the free movement of proteins within the membrane (Singer and Nicolson, 1972). However, this proposed “fluid-disordered status” of membranes has since been revised in favor of a liquid-ordered model: hydrophilic and hydrophobic interactions of lipids with each other result in lipid clustering, promoting ordered structures within the membrane (Simons and Ikonen, 1997; Brown and London, 1998). These microdomains have been termed lipid rafts, because they seem to float in the “ocean” of other lipids (Simons and Ikonen, 1997; Verkade and Simons, 1997; Brown and London, 1998). Compared to most protein-protein interactions, the interactions of lipid molecules with each other are weak and short-lived. Nevertheless, the combination of lipid clustering and lipid-protein interactions enables the lateral sorting of various proteins and thus the formation of dynamic signaling platforms.

**Figure 1:**
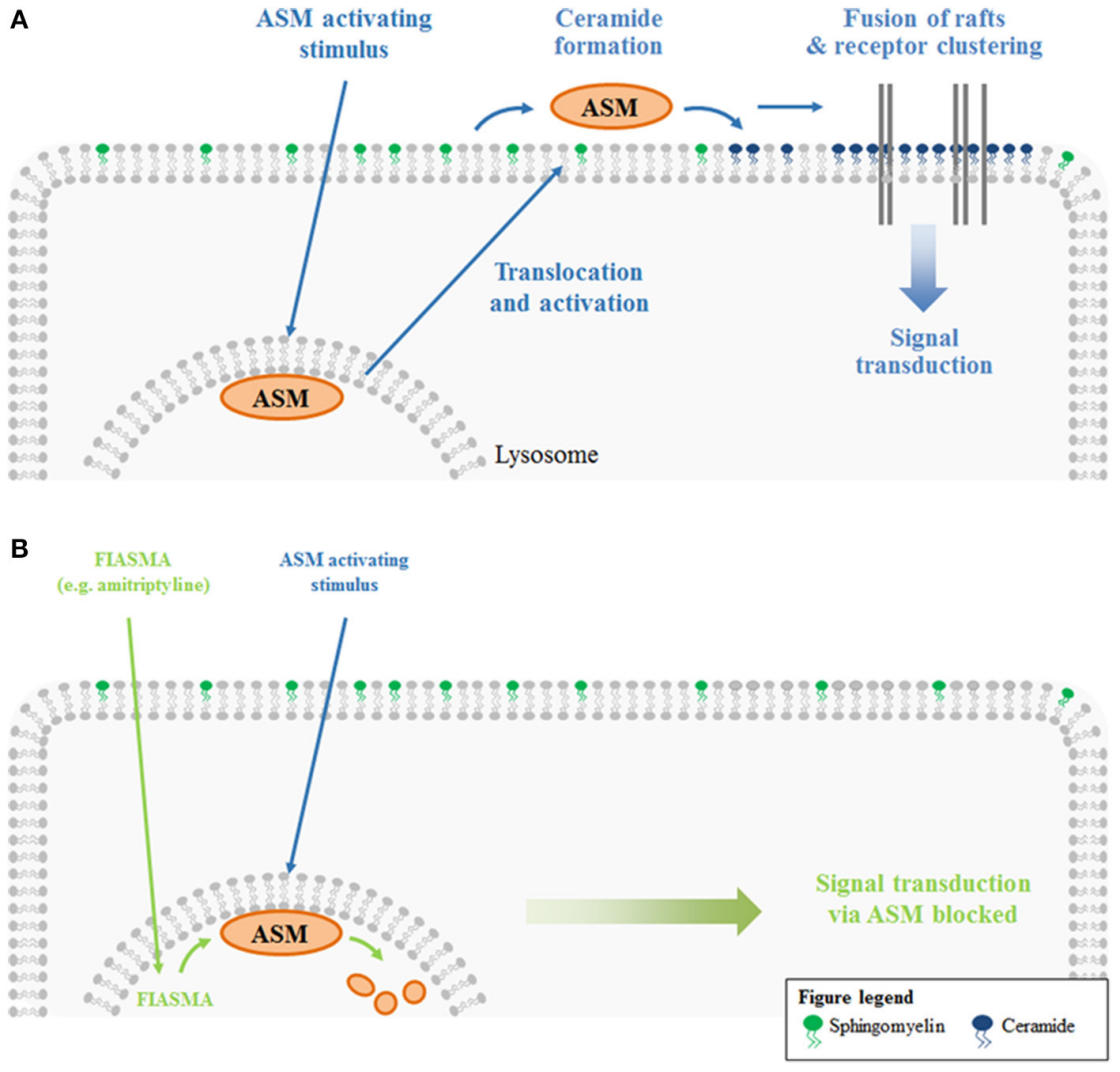
**ASM-mediated platform formation and functional inhibition of ASM. (A)** ASM resides in the lysosome, where it is anchored to the inner lysosomal membrane via electrostatic forces. ASM activating stimuli result in a translocation of the enzyme from the lysosome to the extracellular leaflet of the plasma membrane. There, ASM generates ceramide from sphingomyelin. Due to the self-association of ceramide molecules, ceramide-enriched microdomains are formed. These lipid rafts fuse to large, ceramide-enriched platforms. As a result of lipid-protein interactions, platform formation also results in lateral sorting of proteins. Clustering of specific receptors (and exclusion of others) serves to facilitate and/or amplify signaling processes. **(B)** Functional inhibitors of acid sphingomyelinase (FIASMA) like amitriptyline mediate the lysosomal degradation of ASM. Hence, ASM activating stimuli can no longer induce a translocation of the enzyme to the plasma membrane and the entire signaling cascade downstream of ASM is lost upon amitriptyline treatment.

Ceramide-enriched membrane platforms are one example of lipid domains. Upon certain stimuli, ASM translocates from the lysosomes and secretory lysosomes to the outer leaflet of the plasma membrane (e.g., Grassmé et al., 2001a, 2002; Abdel Shakor et al., 2004; Lacour et al., 2004; Rotolo et al., 2005; Dumitru and Gulbins, 2006; Zhang et al., 2007; Zeidan et al., 2008; Gassert et al., 2009; Perrotta et al., 2010; Avota et al., 2011; Li et al., 2012, 2013). ASM generates ceramide by hydrolyzing plasma membrane sphingomyelin molecules. The generated ceramide molecules associate with each other, forming microdomains. The merging of these small lipid clusters with each other finally results in the formation of large, ceramide-enriched membrane platforms (Grassmé et al., 2001a). The formation of these platforms was shown *in vivo* (Grassmé et al., 2001a) and *in vitro* (Holopainen et al., 1998). The *in vitro* studies revealed that the generation of ceramide is sufficient to trigger the formation of distinct platforms even in purely artificial membranes without any cytoskeleton or other cellular proteins (Holopainen et al., 1998) These platforms selectively trap or exclude specific proteins for biophysical and energetic reasons, and thus serve as a sorting unit for receptors and signaling molecules (Grassmé et al., 2001a, 2003b) (Figure 1A). Some ceramide-interacting proteins are already identified, for instance kinase suppressor of Ras (KSR) (Zhang et al., 1997; Zhou et al., 2002), ceramide-activated protein phosphatase (CAPP) (Dobrowsky et al., 1993; Wolff et al., 1994; Saddoughi et al., 2013), protein kinase C (PKC)-alpha, and -delta (Huwiler et al., 1998), PKC-epsilon (Kashiwagi et al., 2002), PKC-zeta (Müller et al., 1995), c-Raf-1 (Huwiler et al., 1996), phospholipase A_2_ (Huwiler et al., 2001), cathepsin D (Heinrich et al., 1999), inhibitor 2 of protein phosphatase 2A (I2PP2A) (Mukhopadhyay et al., 2009), light chain 3 beta (LC3B-II) (Sentelle et al., 2012). Protein trapping in or exclusion from rafts can facilitate and/or amplify signaling processes. Via this mechanism, ceramide-enriched platforms are involved in many cellular functions like apoptosis, autophagy, inflammation, and senescence (reviewed in Gulbins and Kolesnick, 2003; Gulbins and Li, 2006). Examples are addressed in detail in the third part of this review, which discusses ASM-related diseases. In an effort to treat ASM-related diseases, inhibitors of ASM are necessary. One example for such an agent is amitriptyline, an antidepressant drug.

## Amitriptyline

Amitriptyline is a tricyclic antidepressant (TCA) that was initially introduced by Merck in 1961 for the treatment of major depressive disorder (Merck Sharp and Dohme, 1961). Till today this is the only FDA (Food and Drug Administration)-approved indication, although amitriptyline is already used for a number of other symptoms, including migraine prophylaxis (Mahloudji, 1969; Gomersall and Stuart, 1973; Couch et al., 1976), neuropathic pain disorders (Egbunike and Chaffee, 1990) and fibromyalgia (Carette et al., 1986), nocturnal enuresis (Mishra et al., 1980) and irritable bowel syndrome (Friedman, 1991). Anti-inflammatory and antimicrobial properties of anti-depressive drugs have been reported as well (Roumestan et al., 2007; Mandal et al., 2010).

Like other TCAs, amitriptyline is rapidly absorbed after oral administration (Ziegler et al., 1978; Amsterdam et al., 1980; Brunswick et al., 1980) and extensively metabolized on first-pass through the liver, mainly by cytochrome P450 (CYP450) oxidative enzymes (Bickel and Weder, 1968). N-demethylation of amitriptyline yields nortriptyline, an anti-depressant in its own right. Both amitriptyline and nortriptyline strongly bind to plasma proteins (Borga et al., 1969) and exhibit extensive tissue binding, evidenced by their high apparent volume of distribution (Schulz et al., 1983; Kornhuber et al., 1995; Lombardo et al., 2004). Further metabolization by CYP450 upon hydroxylation and glucoronidation results in inactivation followed by excretion in the urine (Rudorfer and Potter, 1987; summarized in Gillman, 2007).

**Figure 2:**
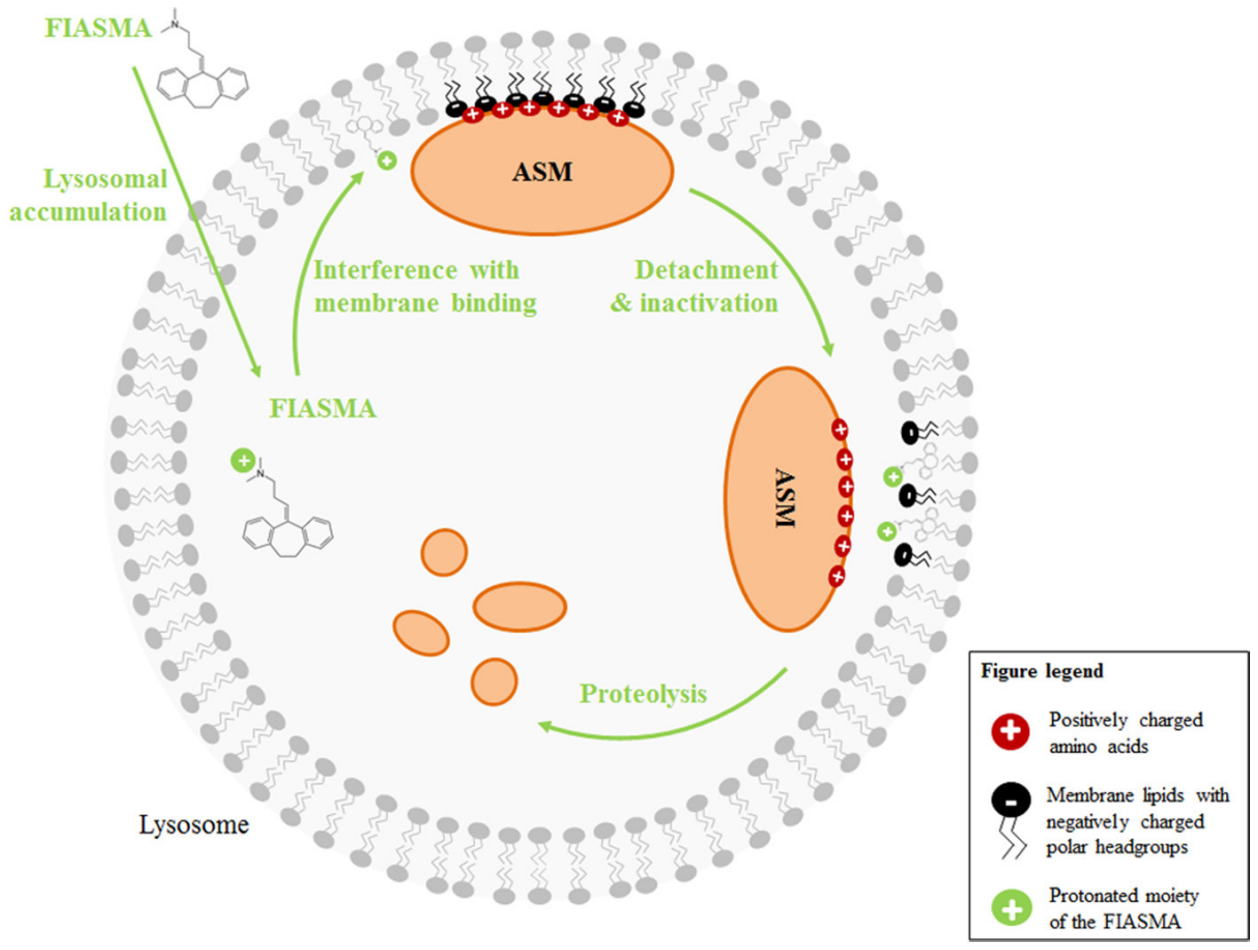
**Mechanisms of action of functional inhibitors of ASM**. FIASMA are weak bases and accumulate in acidic compartments like the lysosome, because they become protonated at the acidic pH. Due to the positive charge, they can then no longer cross the membrane (acidic trapping). While the lipophilic moiety of the FIASMAs is anchored in the lysosomal membrane, the protonated, positively charged portion is exposed to the lumen, thus altering the electrostatic properties of the inner lysosomal membrane. As a result, the electrostatic adherence of ASM to the membrane is lost. Luminal ASM is inactive and targeted by intralysosomal proteases for proteolytic degradation.

Initially, amitriptyline was described as a serotonin-norepinephrine re-uptake inhibitor (Glowinski and Axelrod, 1964) which has a stronger action on serotonin transporters. The metabolite nortriptyline is a more potent and selective norepinephrine reuptake inhibitor (Tatsumi et al., 1997). Additionally, amitriptyline was described to have antihistaminic and anticholinergic properties, because it functions as a receptor-antagonist for a number of histamine and muscarinic acetylcholine receptors (Owens et al., 1997). A number of other receptors are also antagonized by amitriptyline (serotonin receptors, α1-adrenergic receptor), whereas the drug is an agonist for σ 1-, TrkA-, and TrkB-receptors (Owens et al., 1997; Jang et al., 2009). Numerous reports are published describing amitriptyline to block different sodium, calcium, and potassium channels (Schofield et al., 1981; Arita et al., 1987; Wooltorton and Mathie, 1993; Pancrazio et al., 1998; Park et al., 1998; Punke and Friederich, 2007). More importantly, amitriptyline is a functional inhibitor of acid sphingoymelinase (Albouz et al., 1981; Hurwitz et al., 1994b; Kornhuber et al., 2008). The enzyme resides in the lysosome and is usually attached to the inner membrane leaflet by electrostatic forces. Membrane-bound ASM is active and degrades sphingomyelin, yielding ceramide. Administration of antidepressants like amitriptyline or desipramine results in lysosomal accumulation of the drugs (Kornhuber et al., 1995; Daniel and Wojcikowski, 1997). This is due to the weak basicity and high lipophilicity of the drugs, resulting in acid trapping in the lysosome. Weak bases such as amitriptyline passively diffuse across membranes in their neutral state. In acidic intracellular compartments like the lysosome, they become protonated. In this state, they can no longer cross the membrane and thus are trapped inside of the compartment. The accumulation of antidepressants in acidic compartments has been demonstrated experimentally (Ishizaki et al., 1996) and is supported by a single-cell stimulation model (Trapp et al., 2008). The accumulation of antidepressants like amitriptyline or desipramine in the lysosome interferes with the binding of ASM to the membrane, resulting in detachment of ASM and subsequent inactivation by proteolytic degradation (Kölzer et al., 2004). The detached enzyme is a target for intralysosomal proteases and is thus degraded (Hurwitz et al., 1994b; for review see Kornhuber et al., 2014) (Figures 1B, 2).

Weak basicity and high lipophilicity are physicochemical properties amitriptyline shares with other functional inhibitors of acid sphingomyelinase (FIASMAs). These characteristics, rather than specific structural motives, are the prerequisites for ASM inhibition by these drugs (Kornhuber et al., 2008, 2011). The observation that most antidepressants are also lipophilic weak bases may thus explain why so many newly identified FIASMAs are known antidepressants (see Table 2).

**Table 2:** **Tricyclic antidepressants (TCA) and their role as functional inhibitors of acid sphingomyelinase (FIASMA)**.

**TCA (brand name)**	**FIASMA**
Amineptine (Survector)	?
Amitriptyline (Elavil)	yes
Amitripylinoxide (Amioxid)	?
Amoxapine (Ascendin)	?
Butriptyline (Evadyne)	?
Clomipramine (Anafranil)	yes
Demexiptiline	?
Desipramine (Norpramin)	yes
Dibenzepin (Noveril)	?
Dimetacrine (Istonil)	?
Dosulepin (Prothiaden)	?
Doxepine (Sinequan)	yes
Imipramine (Tofranil)	yes
Imipraminoxide (Elepsin)	?
Iprindole (Prondol)	?
Lofepramine (Gamanil)	yes
Melitracen (Adaptol)	?
Metapramine (Timaxel)	?
Nitroxazepine (Sintamil)	?
Nortriptyline (Aventyl)	yes
Noxiptiline (Agedal)	?
Pipofezine (Azafen)	?
Propizepine (Depressin)	?
Protriptyline (Vivactil)	yes
Quinupramine (Kinupril)	?
Tianeptine (Coaxil)	?
Trimipramine (Surmontil)	yes

References: (Kornhuber et al., 2008, 2011).

? = ASM-inhibitory capacity is not yet tested.

An interesting observation was made when using fendiline, a FIASMA previously not known for its antidepressant properties: the authors were able to demonstrate that fendiline had similar antidepressant effects as amitriptyline in a mouse model of major depression (Gulbins et al., 2013). The same study also showed that the antidepressant effect of the tested drugs (amitriptyline, fluoxetine and fendiline) was mediated by the Asm/ceramide system, rather than by a direct effect on neurotransmitter reuptake, leading to neuronal proliferation and survival (Gulbins et al., 2013). It will be interesting to see if more antidepressants will be shown to mediate their effect through ASM inhibition and – on the other hand – if more FIASMAs will be shown to have antidepressant effects.

## Applications of amitriptyline in ASM-related diseases

Since amitriptyline is an efficient ASM inhibitor, it was used for *in vitro* as well as *in vivo* studies to identify the impact of ASM in different diseases. In this context, new roles of ASM and ceramide in biological processes were identified.

### ASM in cardiovascular and metabolic disease

One example for a newly identified role of ASM and ceramide is platelet cell membrane scrambling and thrombus formation: inhibition of ASM with amitriptyline or genetic knock-out inhibited platelet degranulation, phosphatidylserine exposure, and thrombus formation (Münzer et al., 2014). The significance of this, however, still needs further investigation.

Using human cardiac biopsies, Usta et al. (2011) were able to demonstrate that ceramide formation is involved in cardiomyocyte apoptosis, which can occur during cardioplegia and reperfusion. Subjection of the biopsy samples to different cycles of cardioplegia (characterized by blocked perfusion and oxygen supply) and reperfusion *ex vivo* resulted in cardiomyocyte apoptosis in the untreated group. In the amitriptyline-treated samples, apoptosis was significantly reduced. These results suggest that amitriptyline could have a protective effect in ischemia/reperfusion injury in a clinical setting.

According to studies concerning obesity and kidney damage, adipokines (cytokines, which are highly expressed by adipose tissue) mediate the clustering of lipid rafts in glomerular endothelial cells. One adipokine, visfatin, was found to activate ASM, leading to ceramide production and NADPH oxidase activation, thus resulting in increased oxidative stress (Boini et al., 2010a; Xia et al., 2011). This causes disruption of the microtubular network and permeability of the endothelial cell layer (Boini et al., 2010a). Amitriptyline treatment of glomerular endothelial cells not only blocked the formation of ceramide-enriched membrane platforms and release of reactive oxygen species, but also protected the cells from the disruption of the microtubular network – an important aspect of kidney damage (Boini et al., 2010a). These *in vitro* results were verified *in vivo*: analysis of mice on high fat diet revealed elevated ceramide plasma levels, increased adipose tissue Asm activity and higher glomerular damage index (Boini et al., 2010b). Treatment of mice with amitriptyline normalized Asm activity and the plasma ceramide level and protected – at least in part – from cell damage in the kidney. Further analysis showed a faster glucose uptake from the plasma in the amitriptyline-treated group (Boini et al., 2010b). In conclusion, these results indicate that treatment of obesity or metabolic syndrome with amitriptyline could have a protective effect on glomeruli and kidney function.

Ceramide has been implicated to be involved in the pathogenesis of diabetes by mediating beta-cell apoptosis, insulin resistance and insulin synthesis (for review see Galadari et al., 2013). For instance, an increase of skeletal muscle ceramide was observed in obese men with risk for type 2 diabetes and was inversely related to insulin sensitivity (Straczkowski et al., 2007). The role of ASM in diabetes, on the other hand, is not well defined. It was demonstrated that patients with diabetes type 2 show elevated plasma levels of the Zn^2+^-dependent secretory ASM (Gorska et al., 2003) and incubation of rat hepatocytes with ceramide or ASM resulted in the phosphorylation of insulin receptor substrate (IRS-1) leading to blocked insulin signaling and thereby insulin resistance (Herschkovitz et al., 2007). In addition, some common diabetic complications were shown to involve ASM. For instance, ASM was implicated in atherosclerosis (Devlin et al., 2008) and diabetic retinopathy (Opreanu et al., 2010, 2011). Although the mechanism of ASM action in diabetes is not completely investigated FIASMAs are attractive new treatment options for diabetes or at least for these diabetic complications.

### ASM in immune cell function and inflammatory diseases

Xuan and coworkers used amitriptyline to investigate the role of the ASM/ceramide system for the effects of thymol and xanthohumol on the immune system. Thymol is known to have antimicrobial activity (Guo et al., 2009) and xanthohumol was described to have an anticancer effect (Cho et al., 2008). Both studies revealed that incubation of dendritic cells with one of the two chemicals led to ASM activation, ceramide formation and induction of apoptosis by caspase-8 and -3 activation. Thymol- and xanthohumol-induced apoptosis was blocked in ASM-deficient-, as well as in amitriptyline-treated dendritic cells, indicating that ASM and ceramide are crucial for the thymol- and xanthohumol-induced signaling cascade (Xuan et al., 2010a,b).

Dendritic cells were also shown to be sensitive for amyloid-β-peptide- (Aβ_1–42_) and islet amyloid polypeptide (IAPP) induced cell death. Incubation of isolated dendritic cells with these peptides induced ASM activation and ceramide formation, resulting in induction of apoptosis. Pre-incubation of dendritic cells with amitriptyline abrogated this process (Xuan et al., 2010c). These results point to ASM as possible therapeutic target for amyloid-related disease (e.g., Alzheimer's disease) and further studies have to clarify if amitriptyline or analogs can be used to treat these diseases – or at least to ameliorate their symptoms.

With regard to the immune system, Yang and coworkers investigated the role of ASM in mast cell function (Yang et al., 2014). Isolated mast cells from wild type mice or Asm knock-out mice were stimulated with antigens, resulting in a rapid increase of Asm activity in wild type cells, combined with intracellular Ca^2+^ release, activation of K^+^-channels and antigen-induced migration. These effects were abolished or reduced in mast cells from Asm knock-out mice. The reaction of amitriptyline-treated cells to the tested antigens was similar to Asm knock-out cells (Yang et al., 2014). Hence, Asm seems to have an important role in mast cell function and inhibitors of Asm, like amitriptyline, may be potential new anti-allergic agents.

The impact of ceramide on inflammatory bowel disease has also been demonstrated. Analysis of mice with induced chronic colitis revealed an increase of ceramide combined with induction of matrix metalloproteinase 1 (MMP-1) by cytokines like TNF-α and IL-1β (Bauer et al., 2009). Treatment of mice with imipramine, another functional inhibitor of ASM, resulted in blocked MMP-1 induction (Bauer et al., 2009). Because MMP-1 is correlated with destruction of intestinal tissue, the blockage of activation by antidepressants like imipramine or amitriptyline could be a potential therapeutic approach for inflammatory bowel disease.

### ASM in liver diseases

ASM and ceramide were also investigated in Cu^2+^ induced hepatic failure (Wilson disease). In this case, the accumulation of Cu^2+^ was shown to activate ASM. The subsequent ceramide release induced apoptosis of liver cells and formation of cirrhosis (Lang et al., 2007). In a rat model of Wilson disease, amitriptyline treatment protected the animals from cirrhosis, liver failure and early death. Another feature of Wilson disease is anemia. Ceramide was shown to induce the exposure of phosphatidylserine at the plasma membrane of erythrocytes, leading to rapid internalization of the erythrocytes by other cells, thus resulting in anemia. Hence, the authors suggest that pharmacological blockage of ASM may be an effective therapy in Wilson disease patients to protect liver cell death as well as against anemia (Lang et al., 2007).

In terms of hepatic disease, it was also recently demonstrated that Asm inhibition by amitriptyline reduces hepatic fibrosis in mice (Quillin et al., 2014). The authors were able to show that treated mice had significantly less collagen deposits and less activated hepatic stellate cells and an improved hepatic architecture. Thereby it can be concluded that amitriptyline could be an efficient medication for liver fibrosis. Furthermore, a new study by Grammatikos and colleagues show that serum ASM level is significantly upregulated in patients suffering from chronic hepatitis C virus infection or non-alcoholic fatty liver disease (Grammatikos et al., 2014). However, the potential of ASM as a therapeutic target in these diseases has not yet been investigated.

### ASM in major depression

The role of ASM has been extensively studied in major depression. Blood samples from depressed patients exhibited an increased ASM activity in comparison to samples from healthy control subjects. Treatment of peripheral blood mononuclear cells with amitriptyline or imipramine reduced ASM activity and the effect lasted for several days (Kornhuber et al., 2005). Further analysis of the role of ceramide in major depression revealed that amitriptyline and fluoxetine, also a functional inhibitor of ASM, reduce ceramide levels in the hippocampus of mice in a stress-induced depression model. This effect was combined with increased neurogenesis, neuronal maturation and survival of neurons in the hippocampus, as well as normalized behavior (Gulbins et al., 2013). The impact of the ASM/ceramide-system as a potential therapeutic target for the treatment of depressions was recently summarized by Kornhuber et al. (2014). According to their hypothesis, ceramide is formed as a result of stress or infection in the periphery or in the brain itself; causing different effects, e.g., increase of oxidative stress or release of proinflammatory cytokines, and thereby ceramide blocks hippocampal neurogenesis. Reducing ceramide levels should therefore be an efficient way to improve both mood and health in depressed patients.

### ASM in cancer

Lysosomal cell death programs are attractive in cancer therapy because cancer cell lysosomes are less stable than normal lysosomes (Fehrenbacher et al., 2004) and they offer options to circumvent therapy resistance due to defective apoptosis signaling and multi-drug resistance (reviewed in Cesen et al., 2012; Groth-Pedersen and Jäättelä, 2013). In 2013, Peterson and colleagues demonstrated that inhibition of ASM selectively destabilizes cancer cell lysosomes, triggers cancer-specific lysosomal cell death, reduces tumor growth *in vivo* and reverts multidrug resistance (Petersen et al., 2013). Cancer selectivity was associated with a transformation-associated reduction in ASM expression in the tested cells. Cancer cells failed to maintain sphingomyelin hydrolysis during drug exposure to ASM-inhibitors, resulting in lysosomal destabilization due to sphingomyelin accumulation. Hence, some studies have already investigated the effect of ASM-overexpression or use of recombinant acid sphingomyelinase as an adjuvant to conventional chemotherapeutic agents with promising results (Grammatikos et al., 2007; Osawa et al., 2013; Savic et al., 2013). On the other hand a combinatory strategy of ASM inhibition together with conventional chemotherapeutics and/or irradiation could be detrimental, as a number of studies indicate that several chemotherapeutic drugs (Tepper et al., 1999; Lacour et al., 2004; Dumitru et al., 2009a,b) and irradiation (Santana et al., 1996; Paris et al., 2001; Garcia-Barros et al., 2003, 2010; Lee et al., 2011) mediate cell death via ASM activation and ceramide formation. Given these contrasting treatment concepts, it remains to be investigated whether ASM inhibitors can be successfully established as anti-cancer therapeutics.

### ASM in infectious diseases

Ceramide also plays a role in bacterial and viral infections, but also in infectious diseases caused by parasites like *Plasmodia*. In 2008, Brand and coworkers demonstrated that *Plasmodia* require ASM for infection of erythrocytes (Brand et al., 2008). Amitriptyline treatment delayed the increase in parasitemia both in *in vitro* and *in vivo* experiments. Nonetheless, genetic knock-out of Asm in mice did not prolong survival after infection. This might be explained by the finding that *Plasmodium* has its own sphingomyelinase activity and is thus independent of the host's enzyme (Hanada et al., 2002). Brand and coworkers concluded that pharmacological inhibition of ASM by amitriptyline might be a potential strategy to treat malaria, because amitriptyline blocks both parasite- and host sphingomyelinase activity (Brand et al., 2008).

Grassmé and colleagues demonstrated that infection of epithelial cells by rhinoviruses trigger rapid activation and translocation of ASM to the plasma membrane, resulting in the formation of ceramide-enriched membrane platforms, to which rhinovirus then locates (Grassmé et al., 2005). Blockage of ceramide generation by amitriptyline or imipramine, as well as genetic knock-out of Asm prevented rhinovirus infection (Grassmé et al., 2005). Similarly, imipramine was used to investigate the importance of the ASM/ceramide system for ebolavirus infection (Miller et al., 2012). This study demonstrated that virus-like particle require plasma membrane ASM and blockage of ASM inhibited the infection with ebolavirus. Likewise, measles virus (MV) was shown to activate ASM in dendritic cells by ligation of a pattern recognition receptor called DC-SIGN (Avota et al., 2011). DC-SIGN-dependent ASM activation and subsequent formation of ceramide-enriched membrane domains promote the recruitment and clustering of CD150, the uptake receptor for MV, thus promoting virus uptake (Avota et al., 2011). Moreover, an earlier study with MV in T cells demonstrated that accumulation of ceramides in response to MV interfered with the formation of membrane protrusions, T cell spreading, front/rear polarization, and chemokine-induced T cell motility (Gassert et al., 2009). Amitriptyline-treatment rescued lymphocyte cytoskeletal reorganization and thus restored T cell activation and function (Gassert et al., 2009).

In terms of bacterial infections, ASM has a crucial role in infections with a number of pathogens, e.g., *S. aureus*, *L. monocytogenes*, *P. aeruginosa*, *S. thyphimurium*, *E. coli*, and *M. avium* (for review see Grassmé and Becker, 2013). Ceramide-enriched membrane domains were shown to be required for the internalization of *P. aeruginosa*, the induction of death in infected cells and the controlled release of cytokines (Grassmé et al., 2003a). Asm-deficient mice are highly susceptible to pulmonary *P. aeruginosa* infections and infected mice die from sepsis because they are unable to clear the infection (Grassmé et al., 2003a). Similarly, Asm-deficient mice are also highly susceptible to *L. monocytogenes* infections (Utermöhlen et al., 2003): this bacterium is taken up by macrophages and subsequently killed by phagolysosomal fusion. Asm-deficiency prevents this (Schramm et al., 2008; Utermöhlen et al., 2008), enabling the pathogen to escape from the phagosome, replicate in the cytoplasm and spread from cell to cell unaffected by the humoral immune response (Portnoy et al., 1988; Cossart et al., 1989; Goldfine and Wadsworth, 2002; Schnupf and Portnoy, 2007). Furthermore, ASM is also important for the killing of *S. typhimurium* by macrophages via NADPH-mediated release of ROS (McCollister et al., 2007). Therefore, ASM-deficient macrophages were less capable of killing *S. typhimurium* than wildtype cells (McCollister et al., 2007).

The opportunistic pathogen, *E. coli* can cause infectious diseases, including sepsis. Falcone and coworkers demonstrated that ASM-dependent apoptosis of immature dendritic cells in response to *E. coli* contributes to the development of sepsis. ASM inhibition by imipramine prevented dendritic cell apoptosis (Falcone et al., 2004). Next to dendritic cells, endothelial cell apoptosis is a crucial event in LPS-induced endotoxic shock syndrome and ASM was shown to mediate this process (Haimowitz-Friedman et al., 1997). Hence, Asm-deficient mice were protected against LPS-induced endothelial cell apoptosis and thus survived endotoxic shock syndrome (Haimowitz-Friedman et al., 1997). Another example are pathogenic mycobacteria. This class of bacteria typically infects and replicates within macrophages (Oh et al., 1996; Russel, 2001). In infected tissues, the infected macrophages become organized in granulomas together with certain other cells (Russel, 2007). Tissues of Asm-deficient mice infected with *M. avium* contain only small, delimited granulomas and Asm-deficient mice are more resistant to lethal infections with *M. avium* than wild type mice (Utermöhlen et al., 2008).

### ASM in cystic fibrosis and lung injury

Bacterial infections also play an important role in the progression of cystic fibrosis (CF). The disease is caused by a genetic defect in the cystic fibrosis transmembrane conductance regulator (*CFTR*) gene and affects different organs, especially the lungs. Key features of CF lung disease are chronic lung inflammation, recurrent and chronic lung infections and pulmonary fibrosis. Both ASM and acid ceramidase were suggested to be dysregulated in CF. As a result ceramide is extensively generated and accumulates in the lungs of patients. In a mouse model, amitriptyline treatment or partial knock-out of Asm prevented the pathological findings of CF (Teichgräber et al., 2008). Additionally, the ceramide content of bronchial epithelial cells was normalized after inhalation of CF mice with different antidepressants, e.g., amitriptyline, trimipramine, desipramine, fluoxetine, amlodipine, chlorprothixene, or sertraline. Inhibition of Asm also prevented *P. aeruginosa* infection and reduced lung inflammation in CF mice (Becker et al., 2010a). Moreover, amitriptyline treatment also normalized ceramide levels, cell death rates and cytokine release in other tissues affected by CF, e.g., trachea and intestine (Becker et al., 2010b). Recently, it was also shown that ceramide mediates the development of pulmonary fibrosis in CF and that prolonged amitriptyline treatment is able to lessen fibrosis development and to reduce pulmonary inflammatory cytokines (Ziobro et al., 2013).

As a result of these findings, clinical trials were conducted with amitriptyline as a potential medication for CF. In a first randomized cross-over pilot study 4 adult CF patients received amitriptyline for 14 days. This study was followed by a phase IIa study with 19 adult CF patients, who were treated with amitriptyline for 28 days. Amitriptyline was well tolerated in these studies and the treatment significantly improved respiratory function of the patients (Riethmüller et al., 2009). In a subsequent phase IIb randomized, double-blind, and placebo-controlled study, 21 CF patients were treated with amitriptyline for 28 days and compared to 19 patients receiving placebo. Again, an increase of lung function was detected, as well as a decrease of ceramide levels. Safety analysis revealed that amitriptyline did not have any severe effects (Nährlich et al., 2013).

Concerning another inflammatory respiratory condition, Asm was shown to be elevated in a model of acute inflammatory lung disease in neonates (Von Bismarck et al., 2008). The authors hypothesized that Asm may inactivate surfactant and promote proinflammatory responses and thus added imipramine to the surfactant preparation to stabilize it. The addition of imipramine to the exogenous surfactant improved lung function and decreased leukocyte counts and interleukin concentrations in the lavage fluid (Von Bismarck et al., 2008). In a follow-up study, Preuss and co-workers also demonstrated that topical Asm inhibition by imipramine significantly improves several respiratory parameters and lung edema in a triple-hit lung injury model (Preuss et al., 2012). Taken together, these studies make a strong argument for the use of surfactant preparations fortified with an ASM inhibitor for the treatment of neonates with hypoxemic respiratory failure. Additionally, a key player in acute lung injury, platelet-activating factor (PAF) mediates the activation of ASM and the formation of ceramide, resulting in the development of pulmonary edema. Inhibition of Asm by imipramine resulted in a significant reduction of PAF-induced edema in perfused rat lungs (Göggel et al., 2004). Further analysis of the PAF-induced signaling pathway revealed that caveolin-1 is enriched in caveolae of endothelial cells upon ASM activation (Yang et al., 2010). This leads to a decrease of endothelial NO (eNO) formation, which is necessary for the regulation of vascular permeability leading to the formation of pulmonary edema. Pharmacological inhibition of Asm by imipramine blocked PAF-induced decrease of eNO and edema formation (Yang et al., 2010). These results demonstrate a therapeutic approach of ASM-inhibiting drugs to prevent pulmonary edema and acute lung injury.

## Future perspective

Historically, amitriptyline has been employed in the clinic and in research because of its known antidepressant properties. More recently amitriptyline has been investigated because of its capacity to inhibit ASM. In summary, these *in vivo* studies revealed that amitriptyline is a candidate for the treatment of bacterial and viral infections, cystic fibrosis, protection from kidney damage, amelioration of liver cirrhosis and fibrosis and suppression of allergic reactions. An additional, large body of *in vitro* studies expands the potential indications even further – including for instance also Alzheimer's disease. This demonstrates the vast potential of existing drugs for other clinical applications.

Despite the exciting new possibilities for the use of ASM inhibitors, the balance between too much ASM activity and too little (or no) ASM activity is sensitive. One example underlining this is the increased death rate of Asm knock-out mice as a result of bacterial infection, because the bacteria cannot be phagocytosed and killed (Utermöhlen et al., 2003). Another example is thrombus formation due to too little ASM activity in plasma (Münzer et al., 2014). Excessive inhibition of ASM would result in Niemann-Pick disease-like symptoms, whereas insufficient inhibition will result in failure to treat the specific, ASM-related disease. Thus, amitriptyline and all other ASM inhibitors have to be handled with care.

A further reason for caution is the concern about the safety of TCAs like amitriptyline. Due to these concerns, amitriptyline is no longer considered a first-line therapy in depression—even though amitriptyline is at least as effective—if not more—than other antidepressants (Guaiana et al., 2007). TCAs have a wide range of side effects, mostly related to their antimuscarinic properties, e.g., dry mouth, blurred vision, constipation, drowsiness, and dizziness (Potter et al., 2006). Thus, they are often poorly tolerated by patients. Additionally, TCAs have a narrow therapeutic window. To be effective, plasma concentrations of 50–300 ng/ml are required, but toxicity effects start at approximately 450 ng/ml, with major toxicity occurring above 1000 ng/ml and death at about 2000–3000 ng/ml (Schulz and Schmoldt, 2003; Amitai and Frischer, 2004, 2006). The toxic effects are due to ion channel blockade, which results in disruption of cardiac conduction (Marmo et al., 1972). The narrow therapeutic window is a risk in the treatment of depressed patients: amitriptyline, along with other TCAs, is the leading vehicle for completed suicide attempts, accounting for 82% of deaths by antidepressant poisoning. Thus, the continued use as antidepressants has been seriously questioned (Montgomery, 1997).

However, patients suffering from ASM-related diseases that are currently investigated as new amitriptyline targets are not at a higher risk for suicide than the general population. Also, preliminary studies suggest that treatment of these conditions with amitriptyline may require lower doses of the drug than necessary for the treatment of depression and that amitriptyline is also well tolerated at these lower dosages (Riethmüller et al., 2009; Nährlich et al., 2013).

Nevertheless, amitriptyline could potentially also be replaced by other drugs in the treatment of ASM-related diseases. The analysis of further antidepressant drugs tested for their ability to block ASM revealed that other existing drugs are also ASM inhibitors (see Table 2). The search for more inhibitors that specifically block ASM was summarized by Kornhuber et al. (2010). They called this new group of ASM inhibitors FIASMA (Kornhuber et al., 2010). Most of the newly identified FIASMAs are already licensed for the use in humans and are potential alternatives to amitriptyline. The reason why amitriptyline is currently the most widely investigated FIASMA for the treatment of diseases with a deregulation in the ASM/ceramide system is probably that it is the best-known candidate and has been used in the clinic the longest. Moreover, it is easily available and relatively cheap. There is, however, no reason to assume that amitriptyline could not be exchanged in favor of another FIASMA and/or another TCA with better tolerability and safety, should it prove troublesome for one of the new indications in the future. Similarly, future investigations may reveal that other drugs are more efficient or effective than amitriptyline for the treatment of these diseases. For now, however, amitriptyline is a cheap and safe drug with the potential to treat patients suffering from ASM-related disease.

### Conflict of interest statement

The authors declare that the research was conducted in the absence of any commercial or financial relationships that could be construed as a potential conflict of interest.
